# *Fusarium* biocontrol: antagonism and mycotoxin elimination by lactic acid bacteria

**DOI:** 10.3389/fmicb.2023.1260166

**Published:** 2024-01-03

**Authors:** S. Vipin Krishnan, K. Madhavan Nampoothiri, Anandhu Suresh, Nguyen Thuy Linh, P. A. Balakumaran, István Pócsi, Tünde Pusztahelyi

**Affiliations:** ^1^Microbial Processes and Technology Division (MPTD), CSIR-National Institute for Interdisciplinary Science and Technology (NIIST), Thiruvananthapuram, India; ^2^Central Laboratory of Agricultural and Food Products, FAFSEM, University of Debrecen, Debrecen, Hungary; ^3^Department of Molecular Biotechnology and Microbiology, Institute of Biotechnology Faculty of Science and Technology, University of Debrecen, Debrecen, Hungary

**Keywords:** lactic acid bacteria, *Fusarium*, mycotoxin, antagonism, biocontrol bioactive, biocontrol, mycotoxins

## Abstract

Mycotoxins produced by *Fusarium* species are secondary metabolites with low molecular weight formed by filamentous fungi generally resistant to different environmental factors and, therefore, undergo slow degradation. Contamination by *Fusarium* mycotoxins in cereals and millets is the foremost quality challenge the food and feed industry faces across the globe. Several types of chemical preservatives are employed in the mitigation process of these mycotoxins, and they help in long-term storage; however, chemical preservatives can be used only to some extent, so the complete elimination of toxins from foods is still a herculean task. The growing demand for green-labeled food drives to evade the use of chemicals in the production processes is getting much demand. Thus, the biocontrol of food toxins is important in the developing food sector. *Fusarium* mycotoxins are world-spread contaminants naturally occurring in commodities, food, and feed. The major mycotoxins *Fusarium* species produce are deoxynivalenol, fumonisins, zearalenone, and T2/HT2 toxins. Lactic acid bacteria (LAB), generally regarded as safe (GRAS), is a well-explored bacterial community in food preparations and preservation for ages. Recent research suggests that LAB are the best choice for extenuating *Fusarium* mycotoxins. Apart from *Fusarium* mycotoxins, this review focuses on the latest studies on the mechanisms of how LAB effectively detoxify and remove these mycotoxins through their various bioactive molecules and background information of these molecules.

## 1 Introduction

Mycotoxin contamination is one of the primary concerns of all the necessary actions for securing food (Streit et al., 2013). It is predicted that over 25% of global cereal and roughly 20% of global plant production may be contaminated with mycotoxins, and a loss of 60 billion USD is reported due to the same (Johns et al., 2022). However, the level of contamination may vary substantially depending on the regional production and storage environment (Varsha et al., 2014; Bryła et al., 2018). The genera of fungi that can cause mycoses and mycotoxicosis in animals and people are *Aspergillus*, *Penicillium*, *Fusarium*, and *Claviceps*. Long-term mycotoxin exposure has been linked to nephrotoxic, hepatotoxic, immunotoxic, neurotoxic, teratogenic, estrogenic, hemorrhagic, and immunosuppressive effects (Cinar and Onbaşı, 2019). Diverse secondary metabolite mycotoxins like deoxynivalenol (DON), nivalenol (NIV), T-2 and HT-2 toxins, zearalenone (ZEA), fumonisins (FUMs) are produced by *Fusarium* species that are responsible for the production of *Fusarium* mycotoxins usually appear together in contaminated matrices. There are 127 mycotoxin combinations detected up to now, and DON+ZEA, aflatoxins+FUM, aflatoxins+ochratoxin, and FUM+ZEA toxin combinations are the most usual ones (BIOMIN Ltd.).

Various physical, chemical, and enzymatic possibilities exist to eliminate multiple fungal mycotoxins (Karlovsky et al., 2016). Biological methods are available that use microorganisms or their enzymes to mitigate mycotoxins. These strategies can be classified followed by their mode of action: (i) direct or indirect inhibition of the growth and mycotoxin production of *Fusarium* species through; (ii) adsorption of the molecules to polymer cell components, e.g., cell wall constituents; (iii) biotransformation of mycotoxins into non-toxic metabolites; and (iv) conjugation of mycotoxins to cell components.

The ability of bacteria cultures to inactivate and remove *Fusarium* mycotoxins has been discovered and studied for a long time. In recent years, *in vitro* screening helped identify more strains with this specific function, but their reports on food and feed are still limited. This is due to rigorous requests for biological methods applied to the food source of animals and humans. Since safety is a priority, bacteria, besides the detoxification potential, must not contain any unwanted bacterial toxins or allergens, which is why species that have been closely related to food production or are generally GRAS are more favored. In addition, in the case of the detoxification process, the products of the reaction must exert none or less toxicity than their parental molecules and be unable to convert back to the original mycotoxins.

Lactic acid bacteria have an extended history of usage as a natural bio-preservative in food and animal feed (Muhialdin et al., 2020; Petrova et al., 2022). LAB are Gram-positive, non-motile, and catalase-negative bacteria without endospore production, and as their name states excrete lactic acid as a primary product. Homo- and heterofermentative LAB increase the variability of primary metabolites in food and feed products. Diverse antimicrobial substances generated by LAB serve as food preservatives (Shi and Maktabdar, 2022). Additionally, some GRAS LAB strains are chosen as probiotics, which can improve health by immunomodulation and inhibition of internal adhesion of enteropathogenic bacteria (Masood et al., 2011; Arena et al., 2018), working against xenobiotics like mycotoxins (Abbès et al., 2016), or tumor cancer formation (Bell et al., 2022).

This review mainly emphasizes the contribution of the molecular background of LAB antifungal and detoxification potential against mycotoxins produced by *Fusarium* species.

## 2 Diversity of Fusaria

Most plant-pathogenic *Fusarium* spp. are members of four species complexes: *F. fujikuroi* species complex (FFSC), *F. graminearum* species complex (FGSC), *F. solani* species complex and *F. oxysporum* species complex (Aoki et al., 2014). Outside of these four groups, *F. langsethiae*, *F. sporotrichioides*, and *F. poae* are several other species that also receive attention (Munkvold, 2017). Infection of these molds results in a wide range of serious blights, wilts, rots, and cankers, broadly affecting many important crops. Fusarium Head Blight (FHB), the most agricultural concern related to *Fusarium*, has caused huge economic loss, specifically in wheat, barley, and maize production (Wegulo et al., 2015). It is generally induced by members of the *F. graminearum* complex, typically *F. graminearum*, *F. avenaceum*, and *F. culmorum*. Besides FHB, maize production also must face two serious *Fusarium* infection diseases, including Fusarium Ear Rot or “pink ear rot” and Gibberella Ear Rot. While *F. proliferatum*, *F. verticillioides*, and *F. subglutinans*, as members of FFSC, are causal agents of the former, the latter is mainly caused by a few prominent FGSC members: *F. graminearum*, *F. culmorum*, and to a lesser extent, *F. avenaceum* (Ferrigo et al., 2016). The infection is also unavoidable in other crops, additionally causing rot in different parts of the plants and seedling diseases (Broders et al., 2007; Abu Bakar et al., 2013).

Head Blight and other *Fusarium*-caused diseases are known to have a close relationship with the presence of mycotoxins. FHB main carriers, members of FGSC, are potent producers of type A and B trichothecenes (TCTs)—a toxin group that is especially compromised due to its common occurrence and prominent phyto- and myotoxicity, along with other minor pathogens associated with FHB such as *F. poae*, *F. langsethiae* and *F. sporotrichioides* (Munkvold, 2017). ZEA shares some same producers with type B TCTs, with the species of FGSC being the most relevant ones, thus, it frequently co-occurs along with several TCT toxins. Meanwhile, FFSC members, particularly *F. verticillioides* and *F. proliferatum*, are found to be the most important species for FUM production. They predominantly produce type B FUMs, the most frequent group detected, and food and feed. Several new *Fusarium* mycotoxins recently received safety concerns in studies due to their emerging occurrence in plants and crops, including beauvericin, moniliformin, and enniatins (Nazari et al., 2015; Jajić et al., 2019).

In general, moderately warm, and highly moist conditions in this period are favorable for the distribution of most *Fusarium* species. FGSC is frequently found in wheat and barley, with the highest prevalence of *F. graminearum* in many countries of Asia, America, and Europe (Van Der Lee et al., 2015). Its presence is likely observed in cool areas where the mean temperature is not higher than 15°C and is predicted to be distributed in most major rainfed wheat-growing regions of the world (Qu et al., 2007; Backhouse, 2014; Schöneberg et al., 2018). On the other hand, *F. asiaticum* is well adapted to areas where the warmest season has a high mean temperature (>22°C) and precipitation (>320 mm) (Backhouse, 2014). It is reported to prefer rice-growing regions or crops mainly grown in rotation with rice (Van Der Lee et al., 2015). Likewise, *F. langsethiae*, one of the main type A TCT producers, prefers similar conditions but seems slightly more sensitive to water activity than other *Fusarium* species, with no growth observed below 0.90 a*_*w*_*. Its optimal temperature is 25°C and a*_*w*_* > 0.98, representing 28–30% moisture content in grains at 15–25°C, which would be very wet conditions (Medina and Magan, 2010). Under climate change, the alteration in the dominant species and the general abundance in the *Fusarium* complex may also occur (Uhlig et al., 2013). The high occurrence of *F. poae* in wheat is relatively connected to warm and dry conditions during anthesis (Chrpová et al., 2016). *F. poae* and *F. avenaceum* tend to be more active in climatic conditions that are unfavorable for FHB causal agents like *F. graminearum* (Wiśniewska et al., 2014; Covarelli et al., 2015). Meanwhile, *F. boothii* might be sensitive to cold sites but can also be more competitive in warmer regions. It has outnumbered *F. graminearum* to become one of the main *Fusarium* pathogens in wheat in Southern Mexico and Kenya, where it is normally dominant in maize (Cerón-Bustamante et al., 2018). FUM-producing species like *F. proliferatum* and *F. verticillioides* can survive in a wide range of conditions and particularly the most optimal temperature is between 25 and 30°C. *F. verticillioides* additionally show considerably better performance at high temperatures and water stress than *F. proliferatum* (Marín et al., 2010).

## 3 Trichothecenes (TCTs)

**FIGURE 1:**
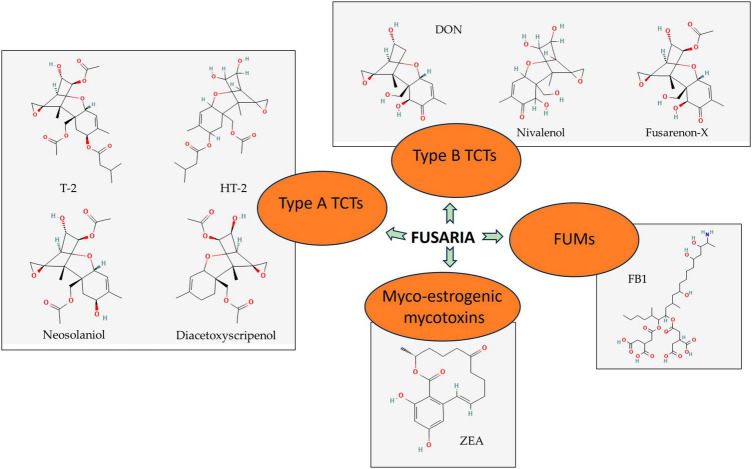
Significant secondary metabolites produced by Fusaria. Type A trichothecenes (TCTs), type B TCSs, myco-oestrogenic mycotoxins, and fumonisins (FUMs) are detected generally in agricultural commodities. Source: National Center for Biotechnology Information (2023). PubChem Compound Summary. Retrieved June 1, 2023.

Trichothecenes consist of a sesquiterpenoid structure, with or without macrocyclic esters or ester ether bridges between C-4 and C-15. The cytotoxicity of a TCT structure is due to the presence of 12, 13-epoxyalkylene groups and 9, 10 double bonds with different side-chain substitutions (McCormick et al., 2011). Type A toxins include T-2 [insariotoxin or 8-(3-methylbutyryloxy)diacetoxyscirpenol], HT-2, neosolaniol (ENNS), and diacetoxyscirpenol (DAS) (Figure 1). Type B toxins include deoxynivalenol (DON) and its derivatives, also nivalenol (NIV), together with the acetylated precursor of NIV [4-acetyl nivalenol, also termed fusarenon-X (FUX)] (Kimura et al., 2007).

Because the target of their producers is greatly diverse, TCTs are found in most of the important economic cereals (e.g., wheat, oat, barley) and legumes (e.g., soybeans), and potatoes during the pre-harvest or post-harvest stage of improperly stored commodities (Delgado et al., 2010; Barros et al., 2011; Bernhoft et al., 2012; Mylona et al., 2012).

Important type B TCTs commonly concerned with food safety are DON and NIV. Their structures only differ in one hydroxyl group, thus sharing various toxicological features. *F. graminearum* and *F. culmorum* are the key fungal species that produce DON. DON is chemically described as 12, 13-epoxy-3α, 7α, 15-trihydroxytrichothec-9-en-8-one (Figure 1). DON acetylated form, particularly 3-acetyl and 15-acetyl-deoxynivalenol (3-ADON and 15-ADON), also catch attention owing to their likelihood of occurrence. These mycotoxins usually accompany the symptoms of FHB and other Fusaria-caused diseases in crops. They show a certain role in the aggressiveness of the pathogens during host invasion since they are one of the *Fusarium* virulent factors (Audenaert et al., 2013). In the case of oral ingestion, intestinal cells are one of the first targets for NIV and DON to conduct function disruption, with the greater acute induced impacts of NIV compared to DON (Ghareeb et al., 2015; Zingales et al., 2021).

Common TCTs of type A are T-2 and HT-2 (Figure 1), identified as secondary products of *F. langsethiae*, *F. poae*, and *F. sporotrichioides*. Diacetoxyscirpenol and neosolaniol (Figure 1) are also under concern, but they have limited incidence. NX toxins—novel TCTs in grains mainly produced by FGSC strains- are also listed in the type A group due to their similar structure (Chen et al., 2022). Despite the higher occurrence frequency and produced amount in food and feed of type B, its toxicity is not comparable to that of type A (Munkvold, 2017).

T-2 is considered one of the most acutely toxic TCTs. T-2 cannot be excreted intact, it is rapidly microbially metabolized in the intestine into HT-2—its major metabolite. However, unlike DOM-1, HT-2 retains nearly completely the toxicity of its precursor (Cope, 2018).

## 4 Myco-estrogenic mycotoxins—Zearalenone and its derivatives

*Fusarium graminearum* and *F. cerealis* are major *Fusarium* species that produce an estrogenic mycotoxin called zearalenone (ZEA) (Figure 1) that contaminates food grains globally. ZEA is 6-(10-hydroxy-6-oxo-*trans-*1-undeceny) β-resorcylic acid lactone, which has a molecular formula of C_18_H_12_O_5_. ZEA and its derivatives are the second most regular *Fusarium* mycotoxins that endanger human health through foods just after TCTs. ZEA has the same producers with type B TCTs, with FGSC being the most important one; thus, it frequently co-occurs along with DON and/or NIV on crops and grains. 17-beta estradiol (17β-estradiol) is a major reproductive hormone resembling the ZEA structure (Zielonka et al., 2014). Unlike TCTs, ZEA neither exhibits fatal acute toxicity toward animals nor is a virulence factor of its producers (Munkvold, 2017). Instead, due to the similarities to estrogenic hormones in chemical structure, it expresses some estrogenic features and primarily targets the reproductive system of animals. After ingestion, ZEA is absorbed and degraded into some of its metabolites, including major ones α-zearalenol (α-ZEA) and β-zearalenol (β-ZEA). Subsequently, they are transferred from the alimentary tract to the reproductive tract, competing with estrogens for binding to estrogen receptors (Zhang et al., 2018).

## 5 Fumonisins

Fumonisins are a group of mycotoxins with a long chain backbone structurally resembling sphingosine. FFSC members, particularly *F. verticillioides*, *F. proliferatum*, and *F. nygamai*, are found to be the main species responsible for FUM production. A series of 4 types of B fumonisins (FBs) named FB1 (Figure 1), FB2, FB3, and FB4 are the most prevalent FUM members detected in food and feed (Li et al., 2015). Among these, FB1 has the highest toxicity and frequency of occurrence, accounting for 70–80% of total FUMs production. Because of the similarities in structure, FB1 expresses its toxicity by disrupting the conversion of sphingosine and inactivating the enzyme ceramide synthase. The congestion leads to the accumulation of free sphingosine and its metabolites and the shortage of desired sphingolipids–important components in eukaryotic cell membrane biology and cellular signaling. In addition, FB1 attempts to imbalance the intracellular dynamic equilibrium between antioxidants and oxidants and regulate gene expression. These series of sabotage are underlying FB1’s immunotoxicity, reproductive toxicity, carcinogenicity, apoptosis, and autophagy initiation (Chen et al., 2021).

FB1 and FB2 are the most common FBs. One of the most representative characteristics of FB1 and FB2 is their long hydrocarbon chain, which contributes to their toxicity (Hussein, 2001). Contaminations by FBs commonly occur during pre-harvest or at the beginning of storage (Marín et al., 2013), and maize is the matrix in which almost all FB contaminations are produced (EFSA, 2014). Contamination levels can vary drastically between maize samples, especially between maize fractions intended for animal feed and raw maize (EFSA, 2014). Despite this high presence of FBs in maize samples, concentration levels do not increase during storage (Marín et al., 2013), which makes it easier to control them.

## 6 Biological control of Fusaria by lactic acid bacteria

Among multiple bacteria species taken into consideration, along with *in vitro* evaluations, LAB and *Bacillus* spp. are two groups of bacteria that are tested on food the most due to their advantages raising the possibility of application. Their role has been reported not to be limited to antimicrobial ability, certain strains could inhibit fungal mycotoxin productions or decrease mycotoxin concentrations of the matrices (Byrne et al., 2022; Nasrollahzadeh et al., 2022a; Raman et al., 2022; Steglińska et al., 2022; Hirozawa et al., 2023; Mateo et al., 2023; Vargas et al., 2023). Not to mention that they can be isolated from food, plants, or animal intestinal and have various applications in both food and non-food sectors. Bacterial cells typically adsorb mycotoxins, such as DON on their cell wall components, or can detoxify these pollutants using enzymes such as ZEA by zearalenone hydrolase. They can simply hinder the growth of molds by different metabolites, such as bacteriocins and acid generation (Soltani et al., 2021; Houssni et al., 2023; Mateo et al., 2023). Arena et al. (2018) claimed that a protonated form of lactic acid inhibits the pH gradient between the outside acidic environment and the inside alkaline cytosol, dissipating the membrane potential and killing cells. LAB metabolites reported that molecules such as hydrogen peroxide, acetate, butyrate, formic acid, succinate, propionate, valerate, caproic acid, and cyclic dipeptides have potent antifungal properties (Fazeli et al., 2004; Bergsma et al., 2022; Zapaśnik et al., 2022). An experiment on sourdough bread between six strains of *Lactiplantibacillus plantarum* showed that strain AR524 at 10^8^ CFU ml^–1^ could remove the highest amount of DON (0.359 mg kg^–1^), and this process majorly occurred in the fermentation stage (Cao et al., 2021). Throughout the simultaneous *in vitro* experiments, neither significant differences were observed for DON reduction rate between viable and non-viable cells, nor DON metabolites were detected. This finding supported pre-existing speculation that the main removal mechanism is physical adsorption by cell wall peptidoglycan and polysaccharides rather than biodegradation. Biotreatment of malting wheat grains with a suspension of bacteria culture containing *Pediococcus pentosaceus* strains greatly affected the mycotoxin content of grains, especially TCT contamination (Juodeikiene et al., 2018; Peter et al., 2022). Instead of exhibiting a direct absorption ability, some strains act as probiotics and reduce the impairment caused by mycotoxins. For instance, *Lacticaseibacillus rhamnosus* RC007 limited intestinal toxicity of DON to pig jejunum explants model at a probiotic dose (García et al., 2018). Since *Bacillus* spp. and *Lactiplantibacillus* spp. are both Gram-positive bacteria with similar cell wall characteristics, ZEA could be absorbed on its surface (Chen et al., 2019; Salah-Abbès et al., 2021). Meanwhile, esterase activity was indicated in certain strains, which is hypothesized to be linked to their ZEA degradation ability by partially contributing to the cleavage of ZEA lactone structure (Chen et al., 2019). Zhao et al. (2015), in a study with the strain *L. plantarum* Lp22 showed a 45 % degradation of ZEA in liquid media through a slow and continuous process by 48 h of incubation.

Recently, more new bacteria strains have been screened *in vitro*, providing more potential options for novel food and feed additives. As previously mentioned, the toxicity of TCTs is mainly determined by the presence of an epoxy ring at C-12, 13 and a double bond at C-9, 10. Hence, biotransformation conducted by several bacteria strains is found to focus on these structures, making TCTs become less toxic agents (McCormick, 2013; Li et al., 2015).

The two main pathogenic fungal strains reported to be suppressed *in vitro* by *P. pentosaceus* are *F. verticillioides* and *F. proliferatum* (Achar and Sreenivasa, 2021). Antifungal activity in *P. pentosaceus* culture supernatant was observed near the end of the exponential growth phase and was pH-dependent. The antifungal metabolites produced demonstrated good thermal stability and resistance to proteolytic enzymes. In culture fractions, substances with molecular weights between 500 and 1400 Da were discovered to have antifungal properties. *P. pentosaceus* has been used frequently in the fermentation of various foods, has GRAS status, and could be a suitable biocontrol organism to improve the quality of ensilage (Magnusson et al., 2003).

### 6.1 Reuterin (3-HPA “complex”)

**FIGURE 2:**
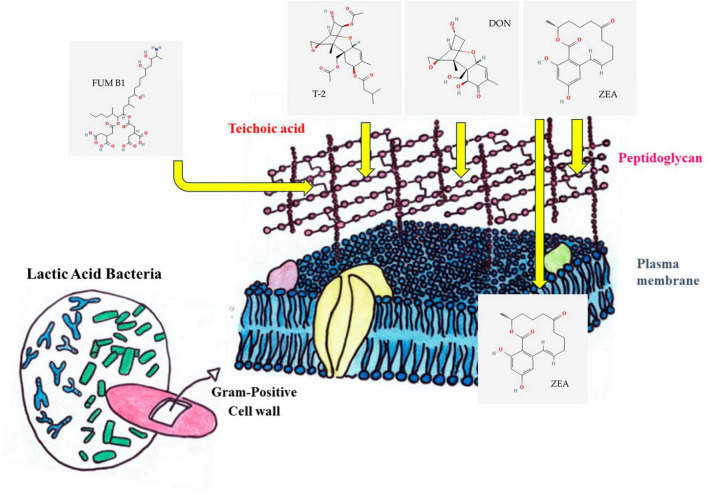
Process of mycotoxin binding. Zearalenone (ZEA) can stabilize hydrophobic and electrostatic interaction with cell surface proteins, adsorption to peptidoglycan, and association with intracellular proteins (Krol et al., 2018). The decrease in T2 and deoxynivalenol (DON) mycotoxins in the environment is also caused by the binding to the cell wall (El-Nezami et al., 2002b). Electrostatic effects and charges on fumonisins (FUMs) direct the toxins toward a unique binding site in the cell wall of LAB. The interaction between FB1 tricarballylic side chain and the peptidoglycan is also well known (Dawlal et al., 2019).

Reuterin is a non-proteinaceous, water-soluble antibacterial compound made by *Limosilactobacillus reuteri*, *L. brevis*, *L. buchneri*, *L. collinoides*, *L. coryniformis* (Magnusson et al., 2003). It has also been generated by other bacterial genera, such as *Bacillus*, *Citrobacter*, *Clostridium*, *Enterobacter*, and *Klebsiella* (Vimont et al., 2019). Under anaerobic conditions, it is produced from glycerol by starving cells (Vollenweider and Lacroix, 2004; Schmidt et al., 2018), and the active reuterin (Figure 2) is not chemically homogenous being a mixture of monomeric, hydrated monomeric, cyclic dimeric forms of 3-HPA (Asare et al., 2019) and acrolein (Engels et al., 2016).

Genes of glycerol dehydratase are usually clustered in the *pdu* operon (Greppi et al., 2019) and it is sometimes also neighboring genes for cobalamin (vitamin B12) synthesis, e.g., *L. reuteri* CRL1089 and organized in *cbi-cob* operon (Morita et al., 2008; Greppi et al., 2019). A study of the complete genome sequence revealed a unique cluster of 58 genes for the biosynthesis of 3-HPA and cobalamin in the reuterin-producing *L. reuteri* JCM 1112T genome both characteristics are beneficial in a probiotic microbe in the intestines. These operons are arranged in the *pdu-cbi-cob-hem* gene cluster (Morita et al., 2008; Greppi et al., 2019).

*Limosilactobacillus reuteri* ATCC 55730 completely lost its 3-HPA-producing ability after five passages of the strains on the same medium under aerobic or anaerobic conditions (Kiňová Sepová and Bilková, 2013). Co-incubation of 3-HPA-producing *L. reuteri* strains with other bacteria like *E. coli* increased the amount of 3-HPA produced. *E. coli-*produced molecules can induce gene expression through quorum sensing (Schaefer et al., 2010) or it is induced by self-induction (Zamudio-Jaramillo et al., 2009).

3-HPA can function in a wide pH range and continue to function in the presence of different enzymes (El-Ziney et al., 1999). It has been mentioned as a potential antibacterial and antifungal agent against a wide range of microorganisms and used as a food preservative in the food industry (Ortiz-Rivera et al., 2017). 3-HPA has been studied for several of its inhibitory properties against a wide range of microorganisms and Fusaria (Rahimi-Kakolaki and Omidi, 2020). Gram-positive microorganisms were more resistant to 3-HPA than Gram-negative microorganisms and *L. reuteri* as a main producer itself has a moderate tolerance against 3-HPA (8.5 mM for *L. reuteri* ATCC 53608) (Ortiz-Rivera et al., 2017). Chung et al. (1989) measured 8.1 mM 3-HPA as a minimal inhibitory concentration MIC for *Fusarium sambucinum*. *L. reuteri* produced reuterin and showed higher activity against fungal spores of *F. culmorum* (*L. reuteri* R29; Schmidt et al., 2018). *F. oxysporum* (*L. reuteri* DSM 20016; Rodrigues et al., 2023).

The research on its antifungal action is limited; however, it was concluded that *L. reuteri* cells are stimulated to produce 3-HPA when are in direct interactions with other microorganisms (Chung et al., 1989). Reuterin is an electrophilic molecule and two possible, but essential pathways can lead to its cytotoxicity (i) by inducing reactive oxygen species (ROS) and (ii) by binding to thiols, modulating selective cysteine oxidation, and causing ribosomal biogenesis inhibition (Bell et al., 2022). Moreover, the reduced form of reuterin, 1,3-propanediol is a volatile compound that increases plant growth (Thomas et al., 2020).

### 6.2 Organic acids

Lactic acid bacteria create a variety of organic acids that are used in a variety of foods and beverages for their antibacterial and flavor-enhancing characteristics. The principal organic acids produced by LAB comprise lactic acid, propionic acid, acetic acid, hydroxyphenyl lactic acid, succinic acid, 3-phenylacetic acid, etc. (Perczak et al., 2018). By lowering the pH in the system, LAB prevent unwelcome microbial and fungal growth. However, most of the fungi prefer slightly acidic medium for their growth, but lower levels of pH attributed to these organic acids are detrimental. To create an acidic cytoplasm, lactic acid diffuses through the cell membrane in a hydrophobic state, breaks down inside the cell, and releases H^+^ ions. For growth and spore germination inhibition of *F. oxysporum f*. spp. *radicis-cucumerinum* all sorbic acid, propionic acid, acetic acid, butylamine, and formic acid had a strong effect (Tzatzarakis et al., 2000). Biological soil disinfestation also applies labile organic acids (acetic, butyric, isovaleric, propionic acids) that were proven to be effective against *F. oxysporum f*. spp. *lycopersici*, *F. spinaciae*, *F. radicis-lycopersici*, and *Fusarium redolens* (Huang et al., 2015). Lactic acid together with citric acid (5% solutions) also lowered the concentration of DON, 15Ac-DON, and NIV, while, citric acid and lactic acid treatments did not affect the concentration of some *Fusarium* mycotoxins, e.g., ZEA, FBs, and culmorin (Humer et al., 2016).

Phenyl lactic acid (PLA) (Figure 2) is a naturally occurring bioactive substance that exhibits broad-spectrum inhibitory activity against a variety of bacteria, fungi, molds, and yeast, including *Enterococcus* spp., *Listeria monocytogenes*, *Salmonella* spp., and *Staphylococcus aureus* (Abdul-Rahim et al., 2021; Wu et al., 2023). The synthesis of PLA in *L. plantarum* was regulated by LuxS/AI-2 quorum sensing mechanism (Chai et al., 2023). LAB like *Lactobacillus*, *Weissella*, and *Leuconostoc* typically produce PLA through the metabolism of phenylalanine. Phenylalanine is transaminated into phenyl pyruvic acid (PPA) in this process, which is then further reduced to PLA by the enzyme lactate dehydrogenase (Guan et al., 2019; Xu et al., 2021). PLA is well known for its potential antifungal activity and additionally, it can prevent asexual reproduction or the condition in fungi by the up-regulation of the *pHiA* gene (Ström et al., 2002; Jung et al., 2019; Xu et al., 2021). PLA is also responsible for the reduction in *Fusarium* growth and decrease of T-2 toxin concentration (Gerez et al., 2009; Kawtharani et al., 2020). The compound can disrupt cell membranes and interfere with mitochondrial energy metabolism (Ning et al., 2021; Zhao et al., 2023). Moreover, PLA does not kill LAB strains, instead, it inhibits their growth and is also harmful to associated pathogens, suggesting that the PLA is the best option for an antimicrobial agent (Guan et al., 2019; Abdul-Rahim et al., 2021) as an alternative to benzoic acid (Ning et al., 2021). As a result, PLA made by LAB increase food product shelf life, enhancing food safety and ultimately improving consumer health. Consequently, PLA has gained widespread acceptance in the food business as a natural preservative.

### 6.3 Cyclic dipeptides (CDP)

**FIGURE 3:**
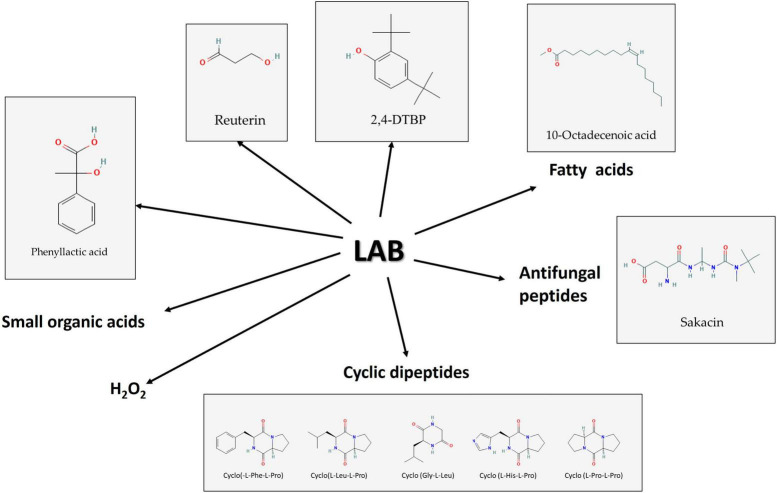
Some of the bioactive molecule groups produced by lactic acid bacteria (LAB) have antifungal effects against Fusaria. Source: National Center for Biotechnology Information (2023). PubChem Compound Summary. Retrieved June 1, 2023.

Cyclic dipeptides are secondary metabolites that have been identified from several bacterial species (Mishra et al., 2017), including *L. plantarum*, and *Bacillus cereus* subsp. *thuringiensis*, and *Bacillus velezensis* AR1 (Bayisa et al., 2021). Higher temperatures do not denature CDPs and are resistant to hydrolytic enzyme denaturation. Individual CDPs are bio-effectors; however, combined CDPs may be bioactive against various diseases. Additionally, the combination of CDPs may display cooperative antibacterial effects whether antibiotics are present or absent. As an antimicrobial activity, CDPs may prevent the synthesis of ergosterol, change the osmotic balance, make the mold membrane porous, reduce mycelial growth, and alter the nuclear DNA’s functional and structural characteristics (Kwak et al., 2014; Sellamani et al., 2016; Nasrollahzadeh et al., 2022b). Dal Bello et al. (2007) found that cyclo(L-Phe L-Pro) and cyclo(L-Leu-L-Pro) produced by *L. plantarum* FST1.7 have antifungal activity against *F. culmorum* and *F. graminearum*. Cyclo(L-Phe-L-Pro) and cyclo(L-Phe-*trans-*4-OH-L-Pro) peptides in synergy with PLA of *L. plantarum* MiLAB 393 were active against *F. sporotrichioides* and *Aspergillus fumigatus* molds, and *Kluyveromyces marxianus* yeast species and are involved in a quorum sensing mechanism (Ström et al., 2002). Cyclo(L-His-L-Pro), cyclo(L-Pro-L-Pro), cyclo(L-Met-L-Pro), cyclo(L-Leu-L-Pro), and cyclo(L-Try-L-Pro) produced by *Lactobacillus amylovorus* DSM 19280 (Ryan et al., 2011), while, cyclo(Gly-L-Leu) (Figure 3) produced by *L. plantarum* VTT E-78076 inhibited the growth of *F. avenaceum* in synergism with lactic acid (Niku-Paavola et al., 1999). *L. lactis subsp. cremoris* produced cyclo(L-Leu-L-Pro) and tetradecanoic acid with antifungal activity (Gajbhiye and Kapadnis, 2021).

### 6.4 Antimicrobial peptides (AMP)

All living organisms produce AMPs, many of which are called antimicrobial peptides because of their relatively small size. Bacteria produce two types of AMPs: synthesized by ribosomes (bacteriocins) and AMPs that are not synthesized by ribosomes, without structural genes. LAB possess peptide transporters that act as a shuttle system to transport the peptides degraded by proteinases into the cell. Intracellular proteinases further cleave the ingested peptides into different amino acids (Zadravec et al., 2022). AMPs are polypeptide fragments with chains ranging in length from 20 to 50 amino acids that have particular antibacterial capabilities against various pathogens and are utilized as biocontrol agents in various industries.

The class of peptides known as antifungal peptides (AFPs) is responsible for the suppression of fungi, and it has been suggested that some LAB can produce AFPs (Chen et al., 2023). According to a study by Coda et al. (2011), a water-soluble antimicrobial peptide produced by a LAB strain can be utilized as a bio-preservative and can inhibit several fungi that are typically found in contaminated bread items. A similar investigation was carried out *in vitro*. The antimicrobial peptide called APT with 40.32 KDa from *L. plantarum* ALAC-4 was discovered to have strong antifungal activity by degrading cell wall and membrane in the pH range of 4-4.5, and the activity was lowered by the proteinase treatment (Chen et al., 2018). It was also found to decrease phosphorus demand and respiratory enzyme activity in *Candida albicans* (Wang et al., 2022).

Bacteriocins are categorized into several classes (Zendo, 2013; Cesa-Luna et al., 2021; Timothy et al., 2021) and LAB are known to produce class IIa bacteriocins which are described as small, heat-stable peptides. Several antifungal bacteriocins were identified along with some organic acids. The development of other microbes, such as bacteria, fungi, and even some viruses, can be prevented by these peptides, which are tiny, cationic molecules and are commonly utilized in fermented foods because they can dramatically reduce the growth of fungi that cause food spoilage and mycotoxin formation (Digaitiene et al., 2012; Garcha, 2018; Woraprayote et al., 2023; Figure 3).

### 6.5 Phenolic compounds

Phenolic compounds are chemicals found in plant systems and associated foods, comprising flavonoids, phenolic acids, and tannins. In comparison to other phenolic compounds, phenolic acids are more extensively investigated because of their diverse antibacterial and antioxidant potential. The hydroxybenzoic and hydroxycinnamic acids make up the two main types of phenolic acids based on their structural characteristics. Gallic, *p*-hydroxybenzoic, vanillic, protocatechuic, and syringic acids are among the most prominent hydroxybenzoic acids, whereas caffeic, *p*-coumaric, ferulic, and sinapic acids are important hydroxycinnamic acids (Martins et al., 2011). Phenolic acids and other organic acids are produced by a variety of microorganisms, and they are efficient food preservation agents against spoilage fungus and mycotoxigenic fungi. A study conducted by Peyer et al. (2016) using barley malt substrate fermented with *L. plantarum* FST1.7 and *L. brevis* R2Δ against *F. culmorum* was found to have significant inhibitory properties. Additionally, the combined action of phenolic acids and organic acids has been demonstrated to be cutting-edge food preservation methods in a variety of food firms. In a recent study, *L. plantarum* and *P. pentosaceus*, which were isolated from olive drupes, showed substantial antifungal activity against *Penicillium nordicum*, *F. oxysporum*, and *Colletotrichum* species. This was due to the presence of 14 different phenolic acids in the fermented broth, which shows that these cultures are effective against the fungi that can contaminate different products made from olives (Riolo et al., 2023). Using chromatographic methods and solvent extraction, the volatile chemical compound 2,4-di-tert-butyl phenol (2,4-DTBP) (Figure 3) was isolated from the cell-free supernatant of *Lactococcus* sp. The chemical was identified as having the molecular structure C_14_H_22_O using ESI-MS, 1H NMR, and FTIR research. It also demonstrated fungicidal efficacy against *F. oxysporum* (Varsha et al., 2014).

### 6.6 Phenazine compounds

Several bacterial species, including some LAB, have been found to produce phenazines, which are nitrogen-containing heterocyclic compounds with antifungal activity. There is limited information on the production of phenazine compounds by Firmicutes, although pyocyanin and phenazine-1-carboxamide (PCA) are examples of phenazine compounds produced by microorganisms (Jayaseelan et al., 2014). Electron microscopic analysis of PCA against *Botrytis cinerea* revealed mycelial withering with edge burrs, rupturing of vacuoles, and distorted structure of various organelles (Zhang et al., 2015). In a phenazine-1-carboxylic acid treatment of *Pestalotiopsis kenyana* phytopathogenic fungus, damage of cell membranes and mycelial formation, reduced mitochondrial membrane potential, and increased ROS levels were observed (Xun et al., 2023). A phenazine compound was obtained from *Lactococcus* spp. BSN307 using solvent extraction and chromatographic procedures (Varsha, 2015). It exhibited a detrimental impact on *F. oxysporum* and has considerable anti-oxidative and anti-cancer properties. More importantly, using molecules from bacteria with a GRAS status is an advantage when using food products directly (Varsha and Nampoothiri, 2016).

### 6.7 Other antifungal compounds

Due to their detergent properties, antifungal fatty acids can destroy the entire plasma membrane and cause the disintegration of the targeted cells by releasing proteins and intracellular electrolytes (Sjögren et al., 2003). From *P. pentosaceus* five active ingredients [9,12-octadecadienoic acid (fatty acid), 3-isobutyl-2,5-piperazine dione (antimicrobial compound), 9,12-octadecadienoic acid (Z, Z), 1-methyl ethyl ester (fatty acid ester), 9-octadecenoic acid (Z) (Figure 3), 2-hydroxy-1-(hydroxymethyl) ethyl ester (hydroxylated fatty acid ester), and pyrrolo[1,2-a]pyrazine-1,4-dione, hexahydro-7-hydroxy-phenylmethyl (a CDP)] were also detected in its antifungal fraction (Ebrahimi et al., 2020).

From the cell-free supernatant of *L. plantarum* MYS6, extracellular metabolites inhibited *Fusarium* growth and FUM production in feed. The major antifungal compounds produced by the isolate were methyl ester; palmitic acid, methyl ester; heptadecanoic acid, 16-methyl ester; stearic acid, and lauric acid besides the recently mentioned 10-octadecenoic acid (Deepthi et al., 2022).

## 7 Enzymatic and physical removal of *Fusarium* mycotoxins

**FIGURE 4:**
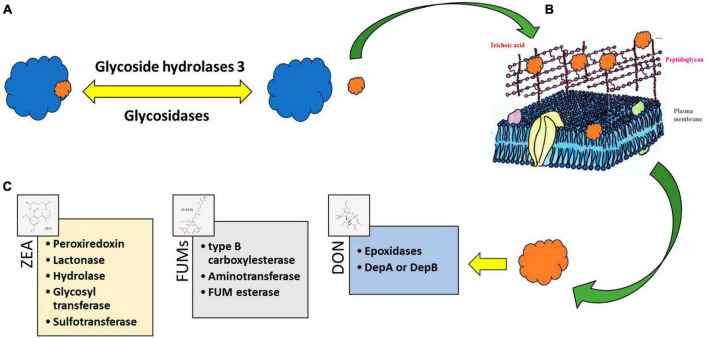
Processes consist of enzymatic modifications like **(A)** glycosidic linkages and their degradation—“freeing” of the plant-bound mycotoxins from a polymer protein or polysaccharide. The free mycotoxin can be adsorbed **(B)** to cell wall structures of lactic acid bacteria (LAB) or can be degraded **(C)** by enzymes. The degradation products become toxicologically inactivated or activated. Source: National Center for Biotechnology Information (2023). PubChem Compound Summary. Retrieved June 1, 2023.

As it was mentioned before, bioactive molecules produced by LAB are known to adopt strategies like adsorption, degradation, or detoxification of mycotoxins to inhibit the growth of *Fusarium* (Średnicka et al., 2021). Some LAB strains ensure the complete removal of mycotoxins through their binding to the cell wall (Vieco-Saiz et al., 2019; Nasrollahzadeh et al., 2022b). The enzymatic or metabolic machinery of LAB degrade or fine-tunes mycotoxins to non-toxins (Li et al., 2020; Nunes et al., 2021; Navale and Vamkudoth, 2022). The diverse composition of LAB cell walls including peptidoglycan, teichoic acids, polysaccharides, and other proteins provides an ambient platform for the adsorption of mycotoxins (Smaoui et al., 2022; Figure 4). The structural diversity of peptidoglycan cell walls and adsorption platforms helps to understand the mycotoxin binding phenomenon of the LAB (Franco et al., 2011; Sadiq et al., 2019). Four major mycotoxins FUM, ZEA, T-2, and DON interaction with cell wall components of LAB and their enzymatic degradation are discussed below.

### 7.1 Zearalenone (ZEA)

Glycoside hydrolase family 3, an enzyme class widely present in bifidobacteria and LAB (Michlmayr and Kneifel, 2014) can help to free the masked, glycosylated ZEA. Adsorption, electrostatic interaction, and ZEA association with inter-cell proteins can happen in ZEA-LAB interactions (Krol et al., 2018). Acid and heat-treated LAB strains, namely GG strain of *L. rhamnosus* and 705 strain, effectively removed α-zearalenol (α-ZOL) through adsorption. Heat-treated GG strain displayed 68% binding affinity toward ZOL and 70% affinity toward ZEA. Acid-treated strain, however, showed nearly equal binding efficiency toward both ZEA and ZOL, respectively. The control strain displayed only 50% binding efficiency toward ZOL and ZEA (El-Nezami et al., 2002a).

*Bifidobacterium* and *L. lactis* exhibited absorption levels at 88 and 90%,respectively (Krol et al., 2018). *L. acidophilus* CIP 76.13T and *L. delbrueckii* subsp. *bulgaricus* CIP 101027T cells removed up to 57% of ZEA (Ragoubi et al., 2021). For *L. paracasei* and *L. lactis*, the maximum sorption effectiveness was 53.3%, and 41.0%, respectively (Rogowska et al., 2019; Złoch et al., 2020).

Likewise, the cell wall of the GG strain of *L. rhamnosus* contains the polysaccharides that play a vital role in the binding of ZEA (Haskard et al., 2000). Polysaccharides of *L. gasseri* contain binding sites for pyrolysates of amino acids (El-Nezami et al., 2004).

Different microbial platforms have been explored for the identification and degradation of ZEA. Through genome engineering, lactonohydrolase from *Rhinocladiella mackenziei* CBS 650.93 (RmZHD) was expressed on *L. reuteri* CGMCC1.3264 surface (Liu et al., 2019) to gain a probiotic and mycotoxin degrading organism. Lactonohydrolase enzyme coded by the gene ZHD101 from *Clonostachys rosea* was expressed in *Kluyveromyces lactis* GG799 (Qiu et al., 2023) and *Escherichia coli* BL21 (Fu et al., 2021). Enzyme characterization clearly showed that the degradation of ZEA was maximum at pH 11 and a turnover number of 0.51 per second. Likewise, enzymes capable of degrading ZEA into multiple small fragments namely ZEA-1 and ZEA-2 were also identified. Peroxiredoxin, lactonase, zearalenone hydrolase, glycosyl transferase, and sulfotransferase were identified as enzymes responsible for ZEA degradation (Lyagin and Efremenko, 2019).

### 7.2 Trichothecenes (TCTs)

Type A TCTs, commonly known as type-2 mycotoxins, are the most toxic among the other TCTs in several foods and feeds. LAB are widely explored in the study of the mechanism of TCT *in vitro*, and most of the mechanism is the binding or attachment to the cell wall (Feinberg and McLaughlin, 2017). According to El-Nezami et al. (2002b), there are significant variations in LAB’ ability to attach TCT to cell walls and remove it from the fermented medium. The experiment conducted by using *L. plantarum* LP102 removed a 19.90 ± 1.70% of T-2 toxin after 24 h of incubation, also further studies revealed that the reduction of T2 toxin was due to the binding to the cell wall of the organism and not due to the bioconversion (Zou et al., 2012). Enzymes such as epoxidase have been reported in the destruction of the epoxy ring in TCTs and related mycotoxins (Boutigny et al., 2008; Awad et al., 2010). Several reports show the presence of the *tri101* gene responsible for the enzyme that does the 3-O-acetylation reaction, which detoxifies trichothecenes via acetylation (Tokai et al., 2005; Manoharan et al., 2006; Garvey et al., 2008).

### 7.3 Deoxynivalenol (DON)

Natural detoxification is mainly carried out by the gastrointestinal microflora and the body metabolism initiating various biotransformation (e.g., de-epoxylation, acetylation, oxidization, etc.). Under the effects of intestinal microbiota, DON can be metabolized into its non-toxic de-epoxidized derivative, de epoxy-deoxynivalenol (DOM-1); hence, the stability and amount of microbiota in the host are quite important to the DON resistance. Polygastric species have a lower susceptibility to these toxins than monogastric animals due to their additional microflora before the colon (Maresca, 2013). In swine, DON quickly presents in their bloodstream due to the fast absorption and little biotransformation, making them very sensitive to DON (Ghareeb et al., 2015). Studies on animals implicated DON as a disruptor of reproductive performance in both genders (Yu et al., 2017).

The ability of the *Lactobacillus* strains to inhibit *F. graminearum* and DON production has been reported. Nearly 51% of DON was eliminated by *L. plantarum* through cell wall binding (Cao et al., 2021). Similarly, a *L. paracasei* strain eliminated 41% of DON through an adsorption mechanism that involved cell wall components. The strain could bind 39% DON and nearly 33% of DON was removed in less than 20 h using a phosphate-buffered solution (Zhai et al., 2019). Nearly, 35% of DON was bound by the genera *Streptococcus* at a concentration of 5 × 10^8^ CFU ml^–1^. *L. acidophilus* CIP 76.13T and *L. delbrueckii* subsp. *bulgaricus* CIP 101027T cells removed up to 30% of DON (Ragoubi et al., 2021).

Enzymes of LAB degrade DON to keto moiety attached DON (DepA) or DepB (Yao and Long, 2020). The Dep A and Dep B enzymes convert DON into DepA (keto form) and further convert it into DepB with less toxicity. The glycosidase enzyme generates the glycosidic acid form of DON to reduce its highly toxic nature. Hydrolysis of oxygen present at the eighth position or acetylation of a hydroxyl group at the third carbon position was reported as another method to reduce the toxicity of DON effectively.

### 7.4 Fumonisins (FUMs)

Peptidoglycans present in the cell wall of LAB are considered to be the central target of FUM. Medium pH was vital in removing fumonisin B1 (FB1) and fumonisin B2 (FB2) by LAB (Niderkorn et al., 2006, 2009). Nearly 80 to 100% of FB1 and FB2 were removed when the pH of the MRS broth was maintained at 4. At neutral pH, the removal efficacy for completely reduced.

Electrostatic effects and charges on FUM direct the toxins toward a unique binding site in the cell wall of LAB. An additional hydrogen bonding with the hydroxyl group (OH^–^) at the 5th carbon and 10th carbon position of FB1 is absent in FB2 or FB3. FB4 displays a different structure with no hydroxyl moieties. Hence, a cage-shaped module formed due to folding interaction between carboxylic acid side chains with backbone modulates the activity of FB1, FB2, and FB3 (Dawlal et al., 2019).

The B7 strain of *L. plantarum* degraded FB1 to nearly 52%, whereas the efficiency increased to 58% using the strain of *L. pentosus* (Zhao et al., 2016). Another report portrayed the distinct role of peptidoglycans of *L. pentosus* for augmented affinity toward FUM (Zhao et al., 2017). Mutants with defective peptidoglycan displayed decreased FB2 only by 20–25% (Niderkorn et al., 2006).

Fumonisins detoxification is mediated by a two-step enzymatic reaction involving de-esterification to produce hydrolyzed FUM followed by deamination. Type B carboxylesterase and aminotransferase were identified as two genes responsible for the degradation of FUMs. Recombinant expression of carboxylesterase and transferase were proved to efficiently catalyze the production of hydrolyzed FB1 and deaminated FB1 in the presence of pyruvic acid (McCormick, 2013). FumD product fumonisin esterase effectively detoxified FB by hydrolysis and removal of the tricarballylic acid groups and was commercialized as FUMzyme^®^ (Alberts et al., 2019, 2021).

## 8 Conclusion

The use of LAB as a biocontrol agent against phytopathogenic fungi presents both challenges and opportunities for the management and storage of food grains and other agricultural products. Food products free of mycotoxins are a major concern in food quality standards. Hence, LAB promise a prominent biological approach in the mitigation of *Fusarium* mycotoxins from food and feedstock, and an appropriate selection and use of LAB strains can be one of the most impacting tools in the control of toxigenic *Fusarium* spp. and their mycotoxins in food. Several bioactive molecules produced by LAB efficiently eliminate *Fusarium* mycotoxin production up to a great extent. Thus, the studies related to the identification and quantification of potent bioactive molecules from LAB contribute greatly to the quality of food grains and this review provides more insight into this based on the latest literature.

## Author contributions

SK: Writing – original draft. KN: Conceptualization, Supervision, Writing – original draft, Writing – review and editing. AS: Writing – original draft. NL: Writing – original draft. PB: Writing – original draft. IP: Conceptualization, Supervision, Writing – review and editing. TP: Conceptualization, Writing – review and editing.
